# Editorial

**Published:** 1902-05-15

**Authors:** 


### EDITORIAL.

The affairs of St. Luke's Hospital, at Niles, Mich., have received considerable attention from some of our dental journals during the past few months, indicating that there is a strong sentiment of antagonism to its methods of conducting its business. There are undoubtedly good grounds for this complaint because of the fact that this institution agrees to issue for a small money compensation a certificate of membership which in form so closely resembles a diploma, that it might be used by unscrupulous persons as such, and thus deceive the public. The institution is not an educational one at all and has no right to issue diplomas and does not undertake to do so. But the form of the certificate comes so perilously near to the form and wording of the ordinary professional diploma that it might readily be taken for such a document. Whether this is intentional on the part of those in charge of the management, or not, would need to be determined by a process of law. The State Dental Society and State Board of Examiners have been asked to take steps to bring this to an issue, but there seem to be no grounds upon which either of these organizations can make a legal complaint. At the recent meeting of the Southwestern Michigan Dental Society a committee was appointed of men living in the vicinity of Niles, to make an investigation and take such steps as may be practicable, either to stop the traffic in this certificate business, or to gather such information as may enable the State society, which meets in June, to take up the matter and do what is possible in enlisting the co-operation of the medical profession and legal authorities in suppressing the illegitimate part of this institution's work at least. Any information that would be of value to this committee, should be forwarded at once to Dr. S. M. White, Benton Harbor, Michigan.

---

COMMENCEMENT season for most of the dental colleges is again at hand, and the question of a location is the one thought which now predominates in the mind of the majority of the graduates. It is also one of some interest to those already settled in practice, as a place must be made for these new graduates, which in some cases means a division of business, or at least some diversion of business to the new man, who comes heralded as the honor man of his class. If not, he makes up for it by equipping his office with all the most modern inventions and beautiful furniture and draperies to be had. In small places professional amenities are often severely strained because of unfriendly attitude of the senior practitioners toward a new comer. There is really no reason for such an attitude in the large majority of instances. There will be room for all the dental graduates for a long time to come, and there are few communities which are over-supplied. Whether this be true or not, the older practitioners are not gainers by omitting a friendly call and a proper recognition of the rights and privileges of the new man. It is better to enter into sympathetic relations than to work at cross purposes.

---

THE proceedings of the National and Southern Dental Associations, have through the enterprise of the *Dental Digest* come to hand just six months after the meeting. This is several months earlier than for any previous meeting. The printing and book making is exceptionally well done, and reflects great credit on the publishers.

